# Building better ECMO rooms: a roadmap to standardization and innovation

**DOI:** 10.1051/ject/2025032

**Published:** 2025-09-15

**Authors:** Nada A. Aljassim, Salman Abdulaziz, John F Fraser

**Affiliations:** 1 Consultant, Pediatric Critical Care Department, ECMO serviceline Lead, Critical Care Services Administration King Fahad Medical City Riyadh Saudi Arabia; 2 Consultant, Cardiovascular Critical Care, Critical Care Services Administration King Fahad Medical City Riyadh Saudi Arabia; 3 Critical Care Research Group Director, University of Queensland & AICS The Prince Charles Hospital Brisbane Australia; 4 Director of Intensive Care Unit, St Andrew’s War Memorial Hospital 457 Wickham Terrace Spring Hill Qld 4000 Australia

**Abbreviations**
ECMO: Extracorporeal membrane oxygenation
ICU: Intensive care unit

Extracorporeal membrane oxygenation (ECMO) is increasingly recognized as a critical intervention for patients with refractory severe cardiac and/or respiratory failure who do not respond to conventional therapies. ECMO is a high-risk and demanding service that requires a trained multidisciplinary team that can provide close monitoring, and precise, thorough, and constant management. Timely intervention is essential in emergencies; any failure to address them when ECMO malfunctions or when a patient deteriorates can lead to life-threatening situations that impact the medical team and can lead to moral distress.

As ECMO use rises globally across all age groups, the need for optimized ICU rooms for ECMO patients – complete with appropriate bed preparation, staffing, and resource allocation – is vital. Such standardization can streamline ICU workflows and enhance patient safety and care quality. Although organizations like the Joint Commission International (JCI) and various critical care societies provide some guidelines for standard ICU setups, there is considerable variability in ICU infrastructure, room layouts, and best practices across different institutions. The set up often relies more on hospital infrastructure, and institutional experience than on established evidence. Furthermore, there are currently no specific evidence-based guidelines for the optimal design of ECMO rooms within ICUs, nor are there studies examining the various setups for running ECMO services and their impact on patient outcomes [1–3]. ICUs with an ECMO specialist model or a perfusionist at the bedside model may have different setups and requirements. Generally, there is no census of where ECMO rooms are located within the ICU or as an ECMO unit in the hospital, but immediate access by the various specialists and caregivers should be guaranteed. It was shown that being within the ICU in a tertiary center is feasible and safe [4, 5].

We summarize the current knowledge on ECMO room preparation, highlight best practices, and propose suggestions that are summarized in Table 1 and shown in Figure 1, for further optimization and potential standardization with future research.

**Figure 1:**
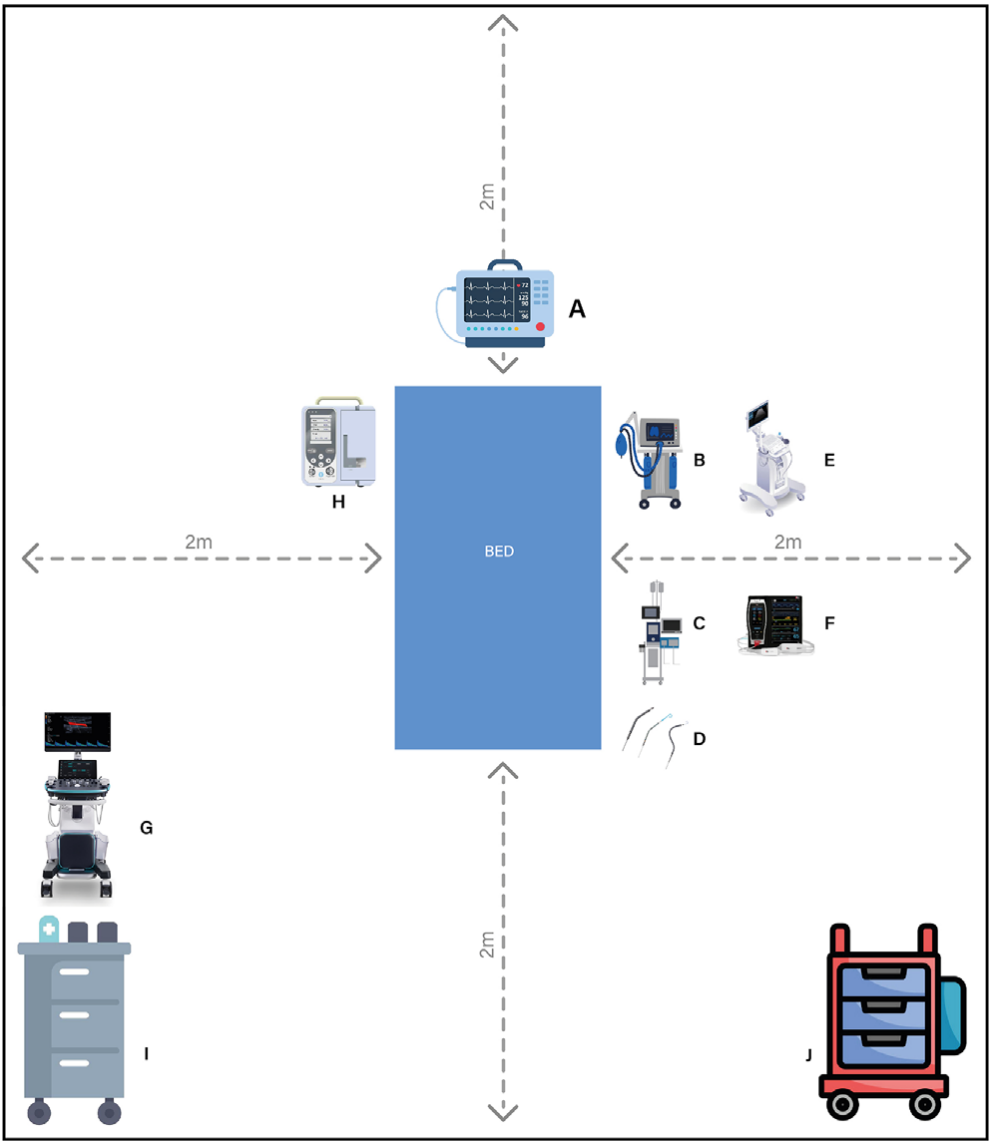
Suggested ECMO room design. Abbreviations: A: patient monitor, B: mechanical, ventilation, C: Impella, D: extra cannula, E: ultrasound, F: Near infrared spectroscopy, H: Intravenous, G: echocardiography, I: medication trolley, J ECMO trolley, m; meters.

**Table 1:** Suggested modifications in the ECMO room.

Category	Suggested details
Space	Room space of at least 30-square meter (sqm) with 200 centimeters (cm) clearance around the bed each side.
ECMO related equipment	ECMO machine (console) + heater/cooler unit, and backup machine. ECMO trolley (clamps, dressing, etc). ECPR back up supply.
Other machines	Mechanical ventilator. Impella. Dialysis or hemofilters.
Monitoring & support equipment	Patient monitor. Blood gas analyzer.
	Point of care coagulation testing like ACT, ROTEM. Noninvasive brain oxymetery.

## ECMO rooms considerations

### Physical space and infrastructure

An optimal ECMO room setup requires careful consideration of ICU layout, space allocation, staff-to-patient ratio, and access to essential equipment for safe and effective care. The literature suggests that a clearance of about 150 cm around the bed should be available, but does not specify the patient type of acuity and the number of machines required [6]. We suggest that patients on ECMO should have more than 25 m of sequence space and at least 150–200 cm of clearance around the bed. In ICUs with few ECMO cases, the ECMO room should be strategically located in a visible area, such as in front of the nurse’s station. In ICUs with many patients on ECMO, all patients should be easily visible to the medical staff. If there are ECMO rooms for patients in isolation, these rooms should preferably have glass doors and be located near an on-call team, including physicians and perfusionists. ECMO rooms, like other ICU rooms, should have minimal interference with noise levels and optimal lighting, which can negatively impact both patients and caregivers [7–9]. The narrative review by Tronstad et al. investigated whether current ICU bed space designs are based on evidence and supportive of patient sleep. The authors found a significant gap between the recommended practices for ICU design and their actual implementation. They suggested optimizing lighting and reducing noise levels to improve patient sleep and overall outcomes. Additionally, a qualitative study underscored the issues associated with suboptimal ICU design [10, 11]. However, high visibility in the ICU was a strong recommendation in the SCCM ICU design guidelines despite the low evidence [2]. Therefore, patients on ECMO are prioritized as critically ill patients on life support and the ECMO rooms should also allow 360-degree access to the patient when needed to facilitate procedures such as cannulation, circuit changes, and emergency interventions. Other general ICU room design recommendations like access to natural light [2]. Further studies to highlight integrating patient and family perspectives into ICU design and compare different or upgraded ICU bed spaces and the environmental factors are crucial to assess different patients’ outcomes, including sleep and circadian Rhythm, delirium, and recovery post-ICU and also improve the patients’ experience [12, 13].

### Equipment organization and readiness

Proper organization and readiness of equipment can significantly reduce response times during emergencies. This includes ensuring standardized placement of the ECMO machine and easy access to emergency supplies like clamps, a backup pre-primed ECMO circuit, and additional cannulas. Also, the ECMO rooms should have an uninterruptible power supply (UPS), and multiple oxygen sources are essential for uninterrupted service, given ECMO’s reliance on power-driven devices.

There are no universal guidelines for equipment layout, but some centers have developed ECMO carts or dedicated stations stocked with necessary supplies. We suggest that the ECMO machine be located at the bedside with the controller (monitor) facing the entrance, allowing visual access to the parameters, easy access to controls, and emergency stop functions [14]. Some centers integrate real-time hemodynamic monitoring with ECMO machine parameters for early complication detection. With advancements in telemedicine, remote ECMO monitoring is becoming feasible. Also, pre-primed ECMO circuits (typically with saline) should be stored in a nearby store for ECMO supply inside the unit or accessible to the team in a short time for emergencies. Other emergency equipment associated with ECMO should be readily available on a dedicated ECMO trolley at the bedside, similar to a crash cart.

New insight and potential innovations

1. Smart ICU design for ECMO patients
  Emerging technologies in ICU design, such as smart alarms, non-invasive telemonitoring, and integrated and automated ECMO monitoring, would enhance patient safety.
2. ECMO-specific ICU units
  Some high-volume ECMO centers have developed dedicated ECMO ICUs with high visibility, are fully equipped, and have staff trained exclusively in ECMO care. The ECMO unit should be designed in proximity to critical hospital areas and required resources, with flexible surge capacity depending on the hospital’s infrastructure and planning of ECMO to be a multidisciplinary or ICU-based approach [3].
3. Tele-ECMO and remote monitoring
  This could allow expert consultation in lower-resource settings and support decision-making in smaller ICUs that lack dedicated ECMO teams [14].

In summary, ECMO rooms and ECMO unit design require careful planning and consideration of various factors to enhance patients’ outcomes and ensure high-quality care. Standardization of practices, adherence to guidelines, and a focus on safety measures are critical for effectively managing patients on ECMO. Future research is required to compare the application of standardized vs. classical ICU rooms design that utilize ECMO, and correlate with ECMO outcomes and patients’ outcomes, including delirium, length of stay, physical weakness, and post-ICU recovery in addition to the engagement of the aligned healthcare on ECMO patients.

## Data Availability

Data available on request.
